# Dental Expenditure by Household Income in Korea over the Period 2008–2017: A Review of the National Dental Insurance Reform

**DOI:** 10.3390/ijerph18083859

**Published:** 2021-04-07

**Authors:** Hosung Shin, Han-A Cho, Bo-Ra Kim

**Affiliations:** 1 Department of Social and Humanity in Dentistry, School of Dentistry, Wonkwang University, Iksan-si 54538, Korea; 2 Department of Dental Hygiene, Shinhan University, Uijeongbu-si 11644, Korea; choruchia@naver.com; 3 Department of Dental Hygiene, Namseoul University, Cheonan-si 31020, Korea; violetbo@naver.com

**Keywords:** National Health Insurance reform, dental expenditures, income-related health inequalities, concentration index

## Abstract

Since 2009, the National Health Insurance in Korea (NHI) has been implementing a series of policies to expand the scope of dental benefits. This study reviewed the changes in co-payments and dental use patterns before (2008 to 2012) and after (2013 to 2017) the NHI’s dental health insurance reform. The study used Korea Health Panel data of 7681 households (16,493 household members) from a 10-year period (2008–2017). Dental expenditures and equivalent income using square root of household size were analyzed. Dental services were categorized into 13 types and a concentration index and 95% confidence interval using the delta method was calculated to identify income-related inequalities by a dental service. Dental expenditures and the number of dental services used increased significantly, while the proportion of out-of-pocket spending by the elderly decreased. The expenditure ratio for implant services to total dental expenditures increased substantially in all age groups, but the ratio of expenditures for dentures and fixed bridges decreased relatively. The concentration index of implant services was basically in favor of the rich, but there was no longer a significant bias favoring the better-off after the reforms. The dental health insurance reform in Korea appears to contribute not only to lowering the ratio of out-of-pocket to total dental expenses per episode in the elderly but also to improving the inequality of dental expenses.

## 1. Introduction

Dental services were covered at a minimum level in the National Health Insurance (NHI) in South Korea. Low-wage households were more likely to fail to meet their dental needs due to financial burdens [1] and were likely to avoid necessary health services when their health needs were the same as those of their counterparts [2]. The excessive burden of health expenditures acted as an economic barrier to the use of dental care, leading to a gap in dental use among income groups [3]. Recently, the National Health Insurance Service has been continuously promoting policies to expand health insurance coverage to alleviate inequality in dental use according to income level [4]. It is also time to discuss the effectiveness of related policies to ensure that dental resources are distributed in an equitable manner [2,5].

Among the factors influencing the use of dental care, income has been discussed in various studies. Duncan and Bonner [6] argued that the need for dental care decreased as income increased. Schwendicke et al. [7] showed that low-income groups had a higher risk of experiencing dental caries than high-income groups. In the low-income class, income served as a factor that prevents receiving dental treatment [8], whereas in the high-income class, perceived barriers such as limited time might act as a barrier to dental care use [9]. Out-of-pocket spending was usually higher in the high-income class, but the low-income class often had higher spending on dental care compared to the high-income class due to poor oral health [10,11].

South Korea, which operates a social health insurance system, has achieved NHI since 1997. A total of 96.3% of the people are covered by the NHI system, and the remaining 3.7% are included in the medical aid system as eligible for basic living security. Prior to the health insurance reform (Table 1), the dental services covered by the NHI were conservative treatment, endodontic treatment, extraction, periodontal treatment including periodontal surgery, and oral surgery. All prosthetic rehabilitation including implants, orthodontic services, and treatments for preventive purposes such as fluoride application and sealant, were out of benefit. The NHI benefit services were inexpensive, but most of the services related to restoring oral health were non-benefit services. For this reason, household income acted as a barrier to dental services among the low-income group, and along with the low coverage rate of the NHI, they had a double burden. According to a study conducted in Korea in 2015, the coverage rate of dental health insurance was 31.9%, which was lower than that of outpatient care (66.0%) and even oriental medicine (47.2%) [4]. In addition, dental implants and orthodontic treatments, which account for the largest expenditure on dental services, were excluded from the aforementioned calculation of the dental health insurance coverage rate; therefore, the dental health insurance coverage rate for the public was expected to be lower than this. Dental health reform was mostly aimed at the elderly, on the basis of reinforcement of welfare policies, starting with complete denture for people aged 75 or older in mid-2012. After that, many policies and plans for strengthening the benefits were actively implemented. 

Previous studies have mostly focused on the factors that affect the use of dental care [6,7,8,9,10,11]. There were a few studies that have investigated the trend of dental expenditures using panel data, but studies with detailed analyses of each dental service were limited. This study examined the trend in spending for each dental service according to income level using the Korea Health Panel and evaluated the effectiveness of policies to expand the coverage of dental insurance by the NHI implemented after 2013 in terms of health inequalities.

## 2. Materials and Methods

This study was conducted with the approval of the Institutional Review Board (WKIRB-201908-SB-066) of Wonkwang University in 2019.

### 2.1. Data

This study used the Korea Health Panel (KHP, version 1.6) data from a 10-year period (2008–2017). The KHP is a household panel survey that employed person-to-person interviews with account books distributed in advance. In order to maintain the representativeness of the nationwide scale, the KHP was built by sampling from the Population and Housing Census using the second-stage probability proportional stratified sampling method. It surveyed 7866 households and 24,616 people nationwide in 2008, and a follow-up survey was conducted every year. The KHP collects individual and household data, such as the socioeconomic characteristics of households and members, income level, living expenses, private medical insurance subscription details, etc., along with the healthcare use, the level of medical expenditure, and health behavior. Using the KHP, it is possible to not only grasp the behavior and changes in healthcare use of the household and individual but also to track changes in healthcare expenses by disease [12]. In this study, 7681 households, 16,493 household members, and 173,863 cases of dental care use were analyzed because of the KHP attrition.

### 2.2. Analyses

The income used in the analysis was gross household income because dental expenditures are more closely related to gross household income than family members’ individual incomes [13]. Gross household income included the sum of ordinary and non-ordinary income, including severance pay, scholarships, gifts, and inheritance [2]. Dental expenditures were analyzed by total dental expenditures and out-of-pocket expenditures. Concentration index (CI) and concentration curves were used to identify income-related dental inequality by income class. The concentration curves for household dental expenditures provide better insight into the distribution of dental expenditures [14].

The CI [15] is widely used in healthcare and health-related research as a tool to measure the inequality of health service use and healthcare cost. In this study, the point estimate CI was calculated using the following equation:

C = 2/*µ* × cov (h,r)

where C is the concentration index, h is the dental out-of-pocket expenditure, and r is the widely accepted square root of household family size equivalence scale. The household equivalence scale reflects the composition of the household and the number of household members to determine the degree of economic well-being of a household unit. Nevertheless, to deal with the issues of the equivalence scale [16], gross household income was used simultaneously with the equivalence scale.

The concentration curve can be located in the upper or lower two areas based on a 45° straight line called the line of equity, and the CI is the area derived by the concentration curve and the equity line. The concentration curve has a maximum value of 1 and a minimum value of -1, where the negative and positive signs indicate the direction of inequality, and the absolute value indicates the degree of inequalities. The 95% confidence interval and p-value of the CI were calculated using the delta method [17,18,19]. If the concentration curve coincides with the equity line, it has a value of 0, and it is interpreted as indicating that there is no income inequality related to the dependent variable.

Dental treatments were classified into 13 groups according to the questionnaire of the KHP: conservative (including amalgam fillings), dentures, fixed bridges, dental implants, orthodontics, periodontics, endodontic, extraction, sealant, tooth whiting, gold inlay, resin filling, and others. Others include oral health education, oral examination, oral surgery, jaw joint treatment, etc. If the treatment is an out-of-coverage in the NHI, dental expenses are often not paid for the same day that treatment is performed. In this case, dental care expenses are paid in several installments. Therefore, there are cases in which the treatment and paid dental expenses do not match. In this case, the total expenses went through a process of redistribution by reviewing the treatment contents for each episode [11]. Here, episode refers to the treatment period in which the patient visits a dental institution and all related treatments are completed.

The study participants were divided into non-elderly and elderly individuals over 65 years of age. In addition, the trend was also examined by the reduction of out-of-pocket expenditures and changes in the behavior of dental care use and expenditures before expanding the dental benefits coverage (2008 to 2012: P1) and after strengthening the benefits (2013 to 2017: P2). Table 1 shows the contents of dental insurance reform over time in South Korea. In the case of denture, the benefit is once in a lifetime, but if the oral condition is seriously changed and it is dentally recognized that new denture is inevitable, a new set denture may be obtained one time. Surveyed crowns of partial denture are limited to porcelain fused to metal crowns, and partial denture using attachments or over-denture using implants are excluded from the benefits. The number of implants is limited to two for a lifetime, and there is no restriction on benefits depending on the disease.

In June 2012, the benefit of complete denture for individuals aged 75+ was first implemented. Since a certain period of time was necessary to access the effectiveness of the policy implementation, partial denture and implants have also been covered by NHI since June 2013. The year 2013 was identified as a suitable time point for evaluating the effect of health insurance coverage. 

Descriptive statistics, such as t-test and chi-square test, were performed to identify the difference of characteristic distribution according to age group (Elderly vs. non-Elderly). The monetary term was converted to purchasing power parity in United States Dollar (PPP USD) [20]. The Difference-in-Difference method, which has been widely used in policy effect analysis, was used to analyze the effect of reducing the co-payments of dental insurance reform using the ratio of out-of-pocket to total dental expenses per episode as the dependent variable (Table S1) [21]. R packages (R version 3.6.3, www.r-project.org) (accessed on 3 June 2020) was used for figures and statistics.

## 3. Results

Among the participants, elderly households and non-elderly households showed different characteristics in all aspects (Table 2). The elderly was more vulnerable than the non-elderly in terms of variables related to dental utilization. The proportion of dental expenses to total outpatient expenditures including dental, medical, and oriental medicine was lower than that of non-elderly households. The low proportion of dental expenses despite the large number of annual dental visits in the elderly group might mean that the proportion of the other expenses due to other systemic diseases was high.

### 3.1. Dental Utilization and Expenditures

**Figure 1:**
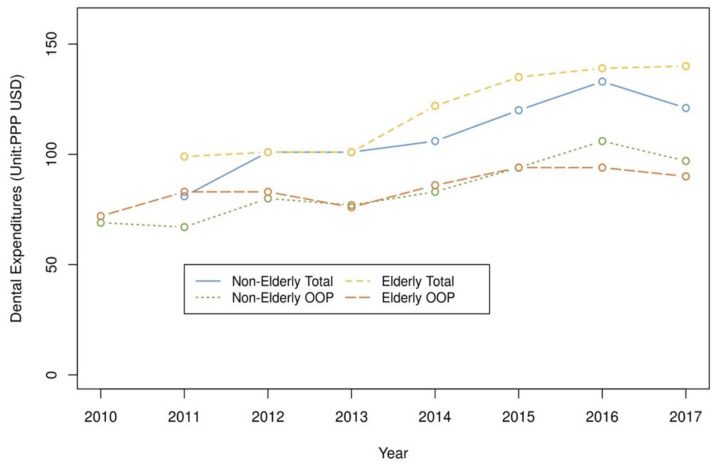
Annual dental expenditures and out-of-pocket spending by age groups. PPP USD: Purchasing Power Parity USD [20]; OOP: Out-of-Pocket.

The KHP’s healthcare expenditure was collected from out-of-pocket (OOP) expenses. Total healthcare expenses, including co-payments and insurers’ contributions, have been investigated since 2011. Total and OOP dental expenditure from 2011 to 2017 and OOP expenses of dental treatments from 2008 to 2017 tended to increase over time (Figure 1).

In the elderly, the total dental expenditures increased, but the OOP expenses did not change significantly, so the proportion of OOP expenses in the total dental expenses visibly decreased (Figure 1). Even after adjusting confounding variables, the OOP proportion to total expenditures of the elderly decreased after dental insurance reform (2013–2017) compared to the previous period (2008–2012) (Table S1). 

**Figure 2:**
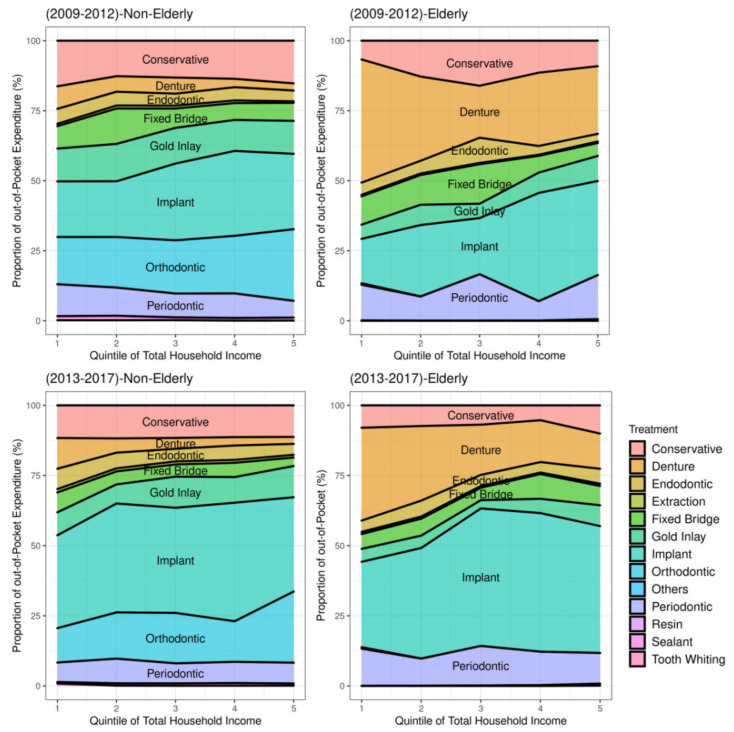
Changes in dental use behavior as a percentage of annual out-of-pocket expenses by period. If the total cost of some treatment areas, such as resin filling, extraction, and tooth whiting, was relatively small, it is not shown in the figure.

There was a distinction in dental utilization behavior by age group and income level. Figure 2 shows the proportion of OOP of each service by income quintile. Overall, in non-elderly, the OOP proportion in dentures, implants, and orthodontics between Q5 (the highest) and Q1 (the lowest) were quite different, and Q5 had a high percentage of dental expenditure in implant and orthodontic services. For the study period, the elderly in Q1 preferred denture, while Q5 had a high proportion of expenditures in implant services.

The implant services showed the largest difference during the period 2013–2017 (P2) compared to 2008–2012 (P1) in both the non-elderly and elderly groups. The proportion of all dental services in the non-elderly, excluding orthodontic and periodontal services, relatively decreased between P1 and P2, while the proportion of implant services increased.

### 3.2. CI and Dental Inequalities

The CI of dental OOP by period and age group was calculated (Table 3). The CI was positive, which means that income-related dental inequalities favored the high-income group. In both P1 (2008 to 2012) and P2 (2013 to 2017) period, the high-income group showed higher OOP expenditures, but the pro-rich inequalities of dental services decreased in P2 compared to P1.

In the CI by dental service, all services except fixed bridge, periodontal treatment, and sealant showed income-related dental inequality in favor of the better-off (Figure S1). 

**Figure 3:**
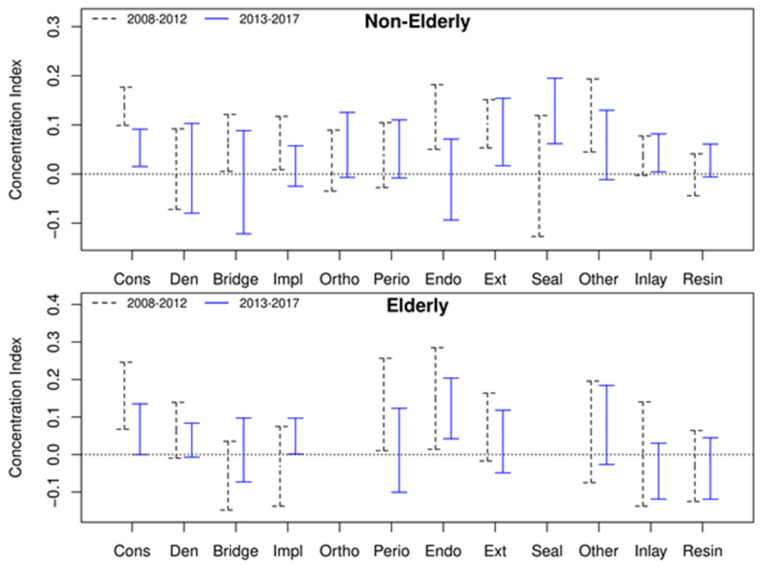
Concentration indices for out-of-pocket expenses of dental treatments by age groups. Cons: Conservative treatment; Den: Denture; Bridge: Fixed bridge; Impl: Dental implant; Ortho: Orthodontic treatment; Perio: Periodontal treatment; Endo: Endodontic treatment; Ext: Extraction; Seal: Sealant; Inlay: Gold inlay; Resin: Resin filling.

Figure 3 showed the changes in the CI index before (2008 to 2012, P1) and after (2013 to 2017, P2) the dental insurance reform. In the case of the elderly, conservative, periodontal, and endodontic services in P1 were inequality favorable to the high-income group, but after the implementation of dental reform (P2 period), this tendency disappeared except for the endodontic service. On the contrary, the CI of dentures was reversed from equality to inequality.

## 4. Discussion

The financial policy of dental public insurance was generally set in the direction of reducing access disparities [22]. Regarding the OOP dental expenditure as a percentage of total dental expenditure in 2011, the average in OECD countries was 55.1%, whereas in South Korea it was 84.2% [23]. The low-income groups had limited access and inadequate use of dental care. This study showed that the financial burden on the elderly decreased due to the influence of the expansion of the NHI’s dental coverage (Figure 1 and Table S1). After the expansion of dental benefits, dental utilization of the lowest (Q1) and the highest (Q5) income group increased by 1.82 times and 1.60 times, respectively, whereas the proportion of the OOP expenditure to household income in Q1 group decreased about 3 times more than that of Q5 (Not presented in the table).

In expanding dental benefits, the service applied to all adults was preventive scaling, not therapeutic scaling, which had already been paid for by the NHI in Korea. For adults aged over 19, preventive scaling once a year has been covered since 2013. The CI of periodontal treatment including scaling and periodontal surgery was positive but not statistically significant. Periodontal disease is a chronic disease that is difficult to manage without conscious effort because there are no specific symptoms unless it worsens to a certain level. For this reason, more resources are spent on periodontal care for higher-income individuals [11]. Prior to the dental reform, the elderly had income-related dental inequality for periodontal services, which were favorable for the high-income group; however, after the policy was implemented, it was changed to not be biased toward either side. Related studies have reported that preventive scaling increased the cost of dental treatment [24,25], but decreased unmet dental needs and increased the number of preventive dental visits [26]. Periodontal disease is one of the main causes of tooth loss as well as the illness associated with major chronic diseases [27]. In this respect, aggravation of periodontal disease in insured persons could cause a financial burden on the NHI and might impede the prevention of many systemic diseases or symptom relief. 

In the elderly, total dental expenditure increased in P1 (2008–2012) compared to P2, whereas OOP expenses remained constant. Those of the non-elderly showed a tendency to increase continuously as time passed (Figure 1). This was evaluated as a result of the benefit expansion of dental health insurance since 2013 (Table S1). During the P2 period (2013–2017), expanding dental insurance benefits such as complete denture, partial denture, dental implants, scaling for prevention, etc., were mostly for the elderly. In the case of the non-elderly, the ease of age restrictions for sealant and the preventive scaling of adults were included for dental benefit packages but had little effect on the reduction of OOP because the total expenses for those treatments were not high.

South Korea’s dental insurance reform started with limited application that applied only to a certain population group, and then extended the applicable age and increased the scope of coverage. For example, the policy for the elderly started with those aged 75 or older and was gradually extended to include those aged 65 and older. The co-payment was also 50% in the beginning, but decreased to 30% in 2019 [28]. Along with the aging society, not only was the number of single elderly households increasing but the income of elderly households was also decreasing [28]. The poverty rate of the elderly over 65 years was high [29], and 22% of the elderly over 65 were not receiving adequate dental treatment, 81% due to economic reasons [30]. The expansion of dental benefit packages would greatly contribute to improving the accessibility of the elderly to dental services.

The study found income-related inequality favoring the better-off in most dental services before dental reform. Conservative services were disproportionately concentrated among the rich because the number of visits per household did not markedly vary by income level, but the average OOP per visit of the high-income group was slightly higher than that of the low-income group. The inequality in favor of the high-income group seen in tooth extraction and endodontic treatment was also the same reason for conservative treatment. That is, the frequency of treatment was high in low-income individuals, but dental expenses per visit were higher among the better-off. Listl [31] identified income-related inequalities in dental service utilization in all countries in Europe. In operative treatment, there was a distribution favoring the better-off in some countries. Geyer et al. [32] also reported that DMFT (decayed, missing, and filled permanent teeth) was 6.06 times higher at the lowest compared to the highest level of income using the Fourth German Oral Health Study. Even within the Japanese public health insurance system, which covered almost all dental services except for some prosthetic benefits, equivalent household income was related to inequality in oral health [33]. Molarius et al. [34] argued that fair access to dental care was important in reducing social inequality in oral health because financially stable groups had better oral conditions; the lower the income was, the more dental care use was avoided. Italian studies also showed that the number of missing teeth, number of decayed surfaces, and number of filled surfaces tended to decrease [35]. In this study, however, after the implementation of dental reform (P2 period), inequality favoring the better-off in most dental services disappeared.

This study identified the distribution of OOP of implant and extraction treatments as services showing income-related inequalities favoring the better-off when calculated regardless of age group (Figure S1). However, as shown in Figure 3, when classified by age group, no income-related inequalities were found. As implant treatment for the elderly has now been included in NHI benefits in 2014 (Table 1), it became an income-related equality service for both the non-elderly and the elderly. Dental implants are covered by public health insurance in some countries [36,37], but implant service in South Korea became the most preferred prosthodontic service. Extraction services by age groups showed no income-related inequality favoring neither the poor nor the rich because extraction services were covered by the NHI, and the service cost was not high; thus, the OOP expenses for patients were small. For example, in the case of the extraction of an impacted tooth, the patient only needed to pay about 35 PPP USD [20]. 

Previous studies have shown variations in the use of dental services according to income brackets. According to the findings of the Korean Community Health Survey, the higher the income level, the higher the dental care utilization for examination [38]. Regular dental visits, check-up visits, periodontal screening, and recording were high in the highest income group [35,39,40]. In the low-income group, the rate of not receiving the treatment recommended by dentists due to cost in prevention and periodontal service was about twice as high as that of the counterpart [41]. In addition to the disparity in the absolute amount of dental expenses, there was also a difference in the relative ratio (ratio of individual service expenditure to total dental expenses) of dental service used by income groups. In this study, the non-elderly Q5 group (the highest income quantile) had a lower percentage of dental expenses than Q1 for denture and periodontal services, but a higher percentage of dental expenses in implant and orthodontic treatment than Q1 (Figure 2). In contrast, in the elderly Q1 group, the denture service was preferred over oral rehabilitation treatment for edentulous mouth, while the implant service expenditure was high in Q5. It was thought that the dependence on the denture service in the elderly relatively decreased as implants were included in the benefit package of the NHI and the cost of implants was reduced (Figure 3). 

The KHP dataset used in this study had some shortcomings. For example, the KHP data were collected via one-to-one interviews. Depending on the level of oral health literacy of participants, their understanding of the treatment received may not have been clear. For these reasons, there might be a difference between the actual treatment received and the 13 categories classified. Through preliminary analysis, observations that might influence the results, for example, services with higher cost than expected, were corrected in the process of data cleaning. In addition, there might be problems with sample representativeness due to panel attrition. However, time and effort were spent maintaining the panel in national agencies (National Health Insurance Service and Korea Institute of Health and Social Affairs). The KHP statistics are still used as crucial data for national policy related to healthcare expenditure in Korea. Accordingly, care must be taken in interpreting the findings.

## 5. Conclusions

This study classified dental services into 13 types and examined the effects of dental health insurance reform policies, focusing on dental utilization behavior and dental expenditures according to income level. In the elderly, the number of dental services used increased significantly, but the ratio of OOP expenses to total dental expenses decreased. Expenditures for implant services increased significantly in all age groups, and the ratio of expenses for dentures and fixed bridges relatively decreased. As a result of the CI of dental services using OOP, distribution of almost all services expenditures, including implants, were no longer favoring the high-income group. In conclusion, the reform of dental insurance since 2013 might be evaluated as a policy that has benefited dental consumers, especially the elderly.

## Figures and Tables

**Table 1:** Expanding dental healthcare coverage of the National Health Insurance.

Time	Newly Added Dental Benefits
December 2009	Sealant (6 to 14 years old) in the 1st molar (sound teeth not affected by caries)
December 2010	Sealant (6 to 14 years old) in the 1st molar (sound tooth with occlusal surface not affected by caries)
July 2012	Complete denture (CD) for people aged 75 or older
October 2012	Sealant in the 1st and 2nd molar teeth of children under 14 years old (sound teeth with occlusal surface not affected by caries)
April 2013	Sealant (under 18 years old) in the 1st and 2nd molar teeth in children/adolescents (sound teeth with occlusal surface not affected by caries)
July 2013	Scaling for all ages (full mouth scaling for prevention, once a year for people over 20 years old)
	Removable partial denture (RPD) for people aged 75 or older
July 2014	Dental implant for people aged 75 or older
July 2015	CD, RPD, and dental implants for people aged 70 or older
July 2016	CD, RPD, and dental implants for people aged 65 or older
August 2017	The co-payment for implants and dentures for people aged 65 and over was reduced from 50% to 30%
October 2017	Sealant’s co-payment rate cut to 10%

**Table 2:** Sample characteristics.

Items	Categories	Elderly (n = 3650)	Non-Elderly (n = 12,843)	*p*-Value ^3^
Age (mean)		73.6	38.2	<0.001
Gender (%)	Male	46.5	43.8	<0.001
	Female	53.5	56.2	
Marital status (%)	Single	30	56.3	<0.001
	Married	70	43.7	
Education (%)	≤Elementary	50.8	24.1	<0.001
	Middle/High	39.6	45.3	
	≥College	9.6	30.6	
Medical aid (%)		6.5	3.0	<0.001
Job (%)	Unemployed	85.2	45.2	<0.001
Equivalence Income ^1^		17,620	30,326	<0.001
Number of annual dental visits	2008–2012	5.00	3.99	<0.001
	2013–2017	5.32	3.81	
Ratio of dental expenditure ^2^ (%)	2008–2012	30.7	46.5	<0.001
	2013–2017	31.1	42.5	

^1^ unit: PPP USD (Purchasing Power Parity Unite States Dollar); ^2^ ratio: dental expenditure to total outpatient expenditures; ^3^ *p*-values were calculated by *t*-test and chi-square test.

**Table 3:** Concentration indices (CI) for dental out-of-pocket expenditures by age group and time period.

	Items	Equivalence Scale			Gross Household Income		
		CI ^1^	95% CI ^2^		CI ^1^	95% CI ^2^	
Total		0.0859	0.0725	0.0993	0.0728	0.0593	0.0864
Age	Non-elderly	0.1011	0.0853	0.1170	0.0903	0.0742	0.1065
	Elderly	0.0802	0.0555	0.1050	0.0728	0.0486	0.0970
Year	2008–2012 (P1)	0.1118	0.0911	0.1326	0.0977	0.0768	0.1185
	2013–2017 (P2)	0.0562	0.0386	0.0738	0.0471	0.0294	0.0648

^1^ CI: concentration index; ^2^ 95% CI: 95% confidence interval.

## Data Availability

Restrictions apply to the availability of these data. Data was obtained from the Korea Institute for Health and Social Affairs and are available at https://www.khp.re.kr:444/eng/main.do (accessed on 3 June 2020) with the permission of the Korea Institute for Health and Social Affairs.

## Supplementary Materials

The following are available online at https://www.mdpi.com/article/10.3390/ijerph18083859/s1, Figure S1: Concentration curves by dental services, Table S1: Difference-in-Difference of out-of-pocket expenses to total medical expenses per episode.
