# Supplementary material for: Dental Expenditure by Household Income in Korea over the Period 2008–2017: A Review of the National Dental Insurance Reform
Source: Int J Environ Res Public Health. 2021 Apr 7;18(8):3859. doi: 10.3390/ijerph18083859 (PMC8067770; doi:10.3390/ijerph18083859)
Supplement: Supplementary file 1 [file ijerph-18-03859-s001.pdf]

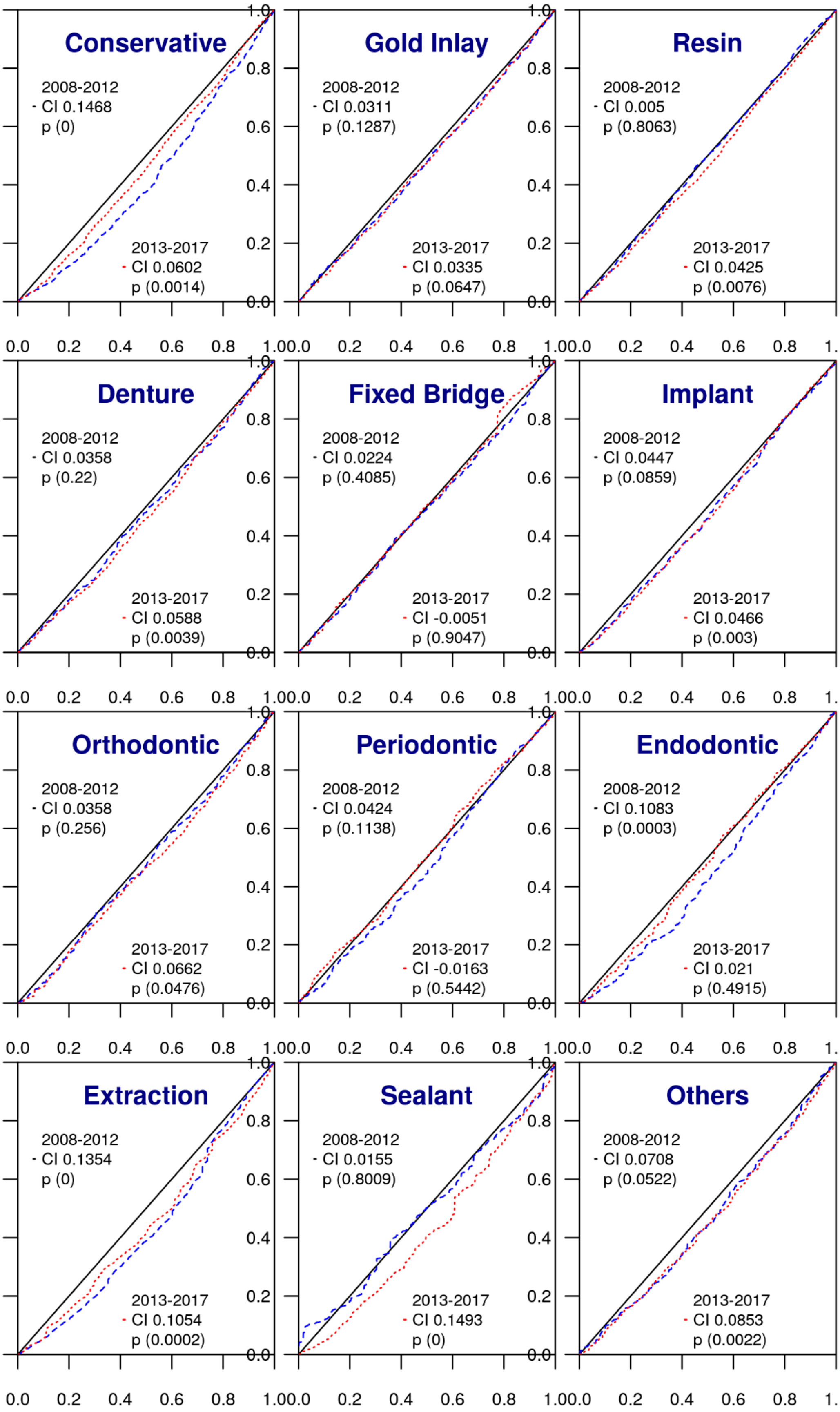

Figure S1. Concentration curves by dental services. CI: Concentration Index; p: p-value.

Table S1. Difference-in-Difference of out-of-pocket expenses to total dental expenses per episode.

Variables	Categories		Coefficients	95% CI[1]		p-value
Period	2008-2012	Ref.				
	2013-2017		-0.005	-0.015	0.004	0.269
Age group	non-Elderly	Ref.				
	Elderly		0.049	0.031	0.068	0.000
Equivalent Income	1st quintile (Q1)	Ref.				
	2nd quintile (Q2)		0.030	0.020	0.041	0.000
	3rd quintile (Q3)		0.043	0.032	0.053	0.000
	4th quintile (Q4)		0.045	0.034	0.056	0.000
	5th quintile (Q5)		0.058	0.047	0.070	0.000
Type of dental treatment	NHI-covered [2]	Ref.				
	Denture & Fixed bridge		0.135	0.127	0.144	0.000
	Gold inlay & Resin filling		0.260	0.242	0.277	0.000
	Implant		0.391	0.371	0.411	0.000
	Orthodontic		0.091	0.082	0.101	0.000
	Tooth whitening		0.135	0.117	0.152	0.000
	Others		0.359	0.349	0.369	0.000
Education	< Elementary school	Ref.				
	Middle/high school		0.060	0.053	0.067	0.000
	> College		0.070	0.061	0.079	0.000
Total number of episodes			-0.016	-0.020	-0.013	0.000
Interaction term[3] (period and age group)			-0.030	-0.049	-0.011	0.002

[1]95% CI: 95% confidence interval; [2]NHI-covered treatments include conservative, periodontics, endodontic, extraction, and sealant.; [3]Interaction term refers to the effect of difference-in-difference, indicating that after the dental insurance reform (2013-2017), the OOP burden in the elderly was lower than in the previous period (2008-2012); Ref: Reference.
